# Ethics in global health research: the need for balance

**DOI:** 10.1016/S2214-109X(15)00095-9

**Published:** 2015-09

**Authors:** Lauren C Ng, Charlotte Hanlon, Getnet Yimer, David C Henderson, Abebaw Fekadu

**Affiliations:** Chester M Pierce MD Division of Global Psychiatry, Department of Psychiatry, Massachusetts General Hospital, Boston, MA 02114, USA (LCN, DCH); Harvard Medical School, Boston, MA, USA (LCN, DCH); Department of Psychiatry (CH, AF) and Department of Pharmacology and Biochemistry (GY), School of Medicine, College of Health Sciences, Addis Ababa University, Addis Ababa, Ethiopia; Centre for Global Mental Health (CH) and Centre for Affective Disorders (AF), Institute of Psychiatry, Psychology and Neuroscience, King's College London, London, UK; and Harvard School of Public Health, Boston, MA, USA (DCH)

Global health research often needs collaboration between various organisations and oversight from many research ethics committees (RECs), including those from partner institutions, national committees, ministries of health, and funders, which increases administrative burden and time. Maintenance of the highest ethical standards in research is paramount; unfortunately ethics breaches are not uncommon.^1^ In view of the real possibility of ethical wrongdoing, worldwide health research must abide by transparent, rigorous, and eff ective procedures of ethics review.

However, although oversight from many RECs might assist with maintenance of ethical principles, this process can result in delays and barriers to research.^2^ Usually each REC reviews protocols independently (either sequentially or in parallel), and will often only review protocols after the other committees have already approved them. Modifi cation requests can be quite different.^2^ Some RECs might request modifications to increase the cultural appropriateness, relevance of the research, and the availability of intervention during and after the project, whereas others might focus on letters of approval and complexity of consent forms.^3^ These competing priorities can mean that previously approved protocols require more amendments, but it is usually unclear which REC's feedback should take precedence.

Having to seek various approvals, with no communication between RECs and no plan for which committee's decisions take priority, can lead to research bottlenecks. Many RECs, particularly those in low-income and middle-income countries (LMICs), have long turnaround times, perhaps because of infrequent meetings, overworked members, and understaffed councils.^2^ These additional demands can make researchers who must show REC approval to apply for grants less competitive for funding. Moreover, the additional administrative burden research teams face to meet the requirements of several RECs might, paradoxically, reduce the time and attention given to the execution of research projects, weakening ethical oversight.

One solution might be for RECs to learn about each other's procedures, communicate about the proposals, and harmonise processes.^4,5^ If a REC could benefit from additional support and capacity building, then collaborating committees could provide this support.

Ideally they could work together to ensure that partner RECs are meeting or exceeding international standards. Collaborative capacity building might be particularly useful for long-term institutional collaborations.^4^ If long-term partnership is not possible, communication between RECs regarding their updated guidelines, submission requirements, expected turnaround times, and agreement about the order of REC submissions might still be beneficial. To help efficient communication between these committees and researchers, we suggest that one REC coordinate feedback and respond to submissions on behalf of all RECs. Whenever possible, the lead REC should be an institutional review board from the country in which the research is being done. If the REC of the institution that is implementing the research is internationally accredited then the final word on approval of, or changes to, the research protocol might be advisably done by that REC.

Another consequence of needing all RECs associated in multinational collaborations (even when research will only be done in one LMIC) to review studies is the reinforcement of the perception that RECs from LMIC do not meet international standards. Although some RECs in LMICs have historically had inadequate ethics training,^6,7^ over the past 20 years, the capacity of RECs from LMIC to do ethics reviews that meet or exceed international standards has been strengthened. Many members of these boards have received ethics training funded or provided by international organisations such as the UN and WHO,^8,9^ and many more are registered with the US Department of Health and Human Services Office for Human Research Protections.^10^ In 2000, 92% of interviewed researchers from LMIC believed that their country's national guidelines for protecting patients involved in research were effective,^3^ a number that would be expected to have increased now because of the high standard of ethical training. Despite these achievements, RECs in LMICs are still sometimes undermined, or are perceived as inadequate or ineffective.

The perception that local RECs are ill-equipped is particularly unfortunate because they might be the most appropriate REC to oversee research projects in their countries. Local RECs are most familiar with the research environment, participant population, and local strengths and challenges. They can also assist with ensuring that sustainable intervention is prioritised and institutional agreements regarding intellectual property rights, ownership of data and samples, and authorship are balanced and equitable. Additionally, some RECs in high-income countries have been perceived as insensitive to local context when reviewing protocols for research in LMICs.^3^ National ethics committees could also assist with overseeing, training, and monitoring the quality of local RECs to meet international standards. If the local RECs are allowed to coordinate all associated RECs and take responsibility for ensuring that all REC feedback is addressed, then the process might be more streamlined and efficient. Additionally, local RECs, which are best positioned to understand the unique contextual ethical challenges in their area, would be empowered to protect the interests of participants and researchers alike.
